# Genome-wide association study and transcriptome analysis reveal key genes affecting root growth dynamics in rapeseed

**DOI:** 10.1186/s13068-021-02032-7

**Published:** 2021-09-10

**Authors:** Keqi Li, Jie Wang, Lieqiong Kuang, Ze Tian, Xinfa Wang, Xiaoling Dun, Jinxing Tu, Hanzhong Wang

**Affiliations:** 1 Oil Crops Research Institute of the Chinese Academy of Agricultural Sciences/Key Laboratory of Biology and Genetic Improvement of Oil Crops, Ministry of Agriculture, Wuhan, 430062 China; 2 National Key Laboratory of Crop Genetic Improvement, Huazhong Agricultural University, Wuhan, 430062 China

**Keywords:** Rapeseed, Root growth, Persistent, Stage-specific, GWAS, WGCNA

## Abstract

**Background:**

In terms of global demand, rapeseed is the third-largest oilseed crop after soybeans and palm, which produces vegetable oil for human consumption and biofuel for industrial production. Roots are vital organs for plant to absorb water and attain mineral nutrients, thus they are of great importance to plant productivity. However, the genetic mechanisms regulating root development in rapeseed remain unclear. In the present study, seven root-related traits and shoot biomass traits in 280 *Brassica napus* accessions at five continuous vegetative stages were measured to establish the genetic basis of root growth in rapeseed.

**Results:**

The persistent and stage-specific genetic mechanisms were revealed by root dynamic analysis. Sixteen persistent and 32 stage-specific quantitative trait loci (QTL) clusters were identified through genome-wide association study (GWAS). Root samples with contrasting (slow and fast) growth rates throughout the investigated stages and those with obvious stage-specific changes in growth rates were subjected to transcriptome analysis. A total of 367 differentially expressed genes (DEGs) with persistent differential expressions throughout root development were identified, and these DEGs were significantly enriched in GO terms, such as energy metabolism and response to biotic or abiotic stress. Totally, 485 stage-specific DEGs with different expressions at specific stage were identified, and these DEGs were enriched in GO terms, such as nitrogen metabolism. Four candidate genes were identified as key persistent genetic factors and eight as stage-specific ones by integrating GWAS, weighted gene co-expression network analysis (WGCNA), and differential expression analysis. These candidate genes were speculated to regulate root system development, and they were less than 100 kb away from peak SNPs of QTL clusters. The homologs of three genes (*BnaA03g52990D*, *BnaA06g37280D,* and *BnaA09g07580D*) out of 12 candidate genes have been reported to regulate root development in previous studies.

**Conclusions:**

Sixteen QTL clusters and four candidate genes controlling persistently root development, and 32 QTL clusters and eight candidate genes stage-specifically regulating root growth in rapeseed were detected in this study. Our results provide new insights into the temporal genetic mechanisms of root growth by identifying key candidate QTL/genes in rapeseed.

**Supplementary Information:**

The online version contains supplementary material available at 10.1186/s13068-021-02032-7.

## Introduction

Rapeseed (*Brassica napus* L*.*; Brassicaceae), a globally cultivated crop, is not only one of the essential vegetable oil sources, but also an important emerging biodiesel and biofuel sources for industrial production [1]. Currently, biodiesel is mainly made from the monounsaturated fatty acids from vegetable oils [2]. Rapeseed oil has the highest percentage of monounsaturated fatty acids among the plant oils. Biodiesel has been manufactured primarily from rapeseed oil in Europe [3]. Rapestraw can be used to produce liquid biofuel, particularly ethanol, since it contains abundant lignocellulosic material [4]. Therefore, it is necessary to boost rapeseed biomass and yield so as to satisfy the increasing demand for edible oil and fuel worldwide.

The root system architecture (RSA) usually denotes the spatial configuration of complex assembly of the root system, and root shape plays key role in healthy plant growth, since root system penetrates the soil in search for water and nutrients [5]. Therefore, plants rely on the modulation of RSA in response to a changing soil environment to increase yield potential and yield stability. The genetic improvement of root architecture, such as increasing lateral root (LR) number, facilitates resource bioavailability in plants and increases crop yield and stress tolerance [6, 7]. To breed the crops with better RSA, a large number of studies have focused on variations in root architecture in many crops, such as rice, wheat, maize, soybean, and rapeseed [8–12]. Several studies have identified hundreds of root QTL in controlled environments or in the field [11, 13–15]. Besides, several of these QTL have also been reported to influence such traits as yield, water/nutrient uptake, and abiotic stress tolerance [13, 16–21].

Genome-wide association study (GWAS) has been successfully used for the identification of the polymorphism sites and/or genes related to complex traits including root traits in crops, such as rice, wheat, maize, and rapeseed [10, 14, 15, 22, 23]. The *Brassica* 60 K Illumina single-nucleotide polymorphism (SNP) array has facilitated the genetic improvement of different traits including flowering time, seed oil content, and phosphate-efficiency to obtain desirable alleles in *B. napus* [14, 24, 25]. Moreover, *brassinosteroid signaling kinase 3* (*BSK3*) was confirmed to regulate root elongation at the low-nitrogen condition in Arabidopsis by GWAS [26]. Transcriptome analysis has become an effective technique for detecting candidate genes. Many crucial differentially expressed genes (DEGs) related to root development have been identified by RNA sequencing in rice, maize, and *B. napus* [27–29]. Weighted gene co-expression network analysis (WGCNA) has been usually used to analyze the relationship and network between different genes. Functional candidate genes related to root development were identified at different developmental stages in crops by WGCNA, including *DcMYB113,* which was reported to regulate anthocyanin transport in carrot root [30], and three hub genes (*GRMZM2G477658*, *GRMZM2G15536*, and *GRMZM2G072121*) played a possible role in maize root formation and growth through the division and/or elongation of cells [31]. Recently, the combination of GWAS, transcriptome sequencing, and/or WGCNA has been turned out to be a rapid and efficient approach to identifying crucial candidate genes regulating root development [9, 15, 32, 33]. For example, *OsNal1* and *OsJAZ1* located in the peak SNPs have been confirmed to facilitate the root development in rice [33].

Root growth is a continuous and complex process with temporal dynamics and spatial patterning. A previous study has defined seven root growth types in a *B. napus* recombinant inbred line (RIL) population derived from two rapeseed cultivars (Zhongshuang11 and NO. 73290) with contrasting root systems, and identified two types of QTL (persistent and stage-specific) by the analysis of root traits in rapeseed [11]. To further identify the genetic factors controlling the dynamic root growth, we examined five continuous stages during root development in 280 natural accessions of *B. napus*. Sixteen persistent and 32 stage-specific QTL clusters further confirmed the existence of the two types of QTL controlling root development. In addition, we performed a transcriptome analysis of samples of four root growth types with extremely contrasting growth rates during the investigated timepoints. A total of 12 crucial candidate genes involved in root growth were identified via combining GWAS, WGCNA, and differential expression analysis, some of which have been reported to be related to root development in previous studies.

## Results

### Phenotypic analysis of 280 *B. napus* accessions reveals genetic stability of root development

**Fig. 1:**
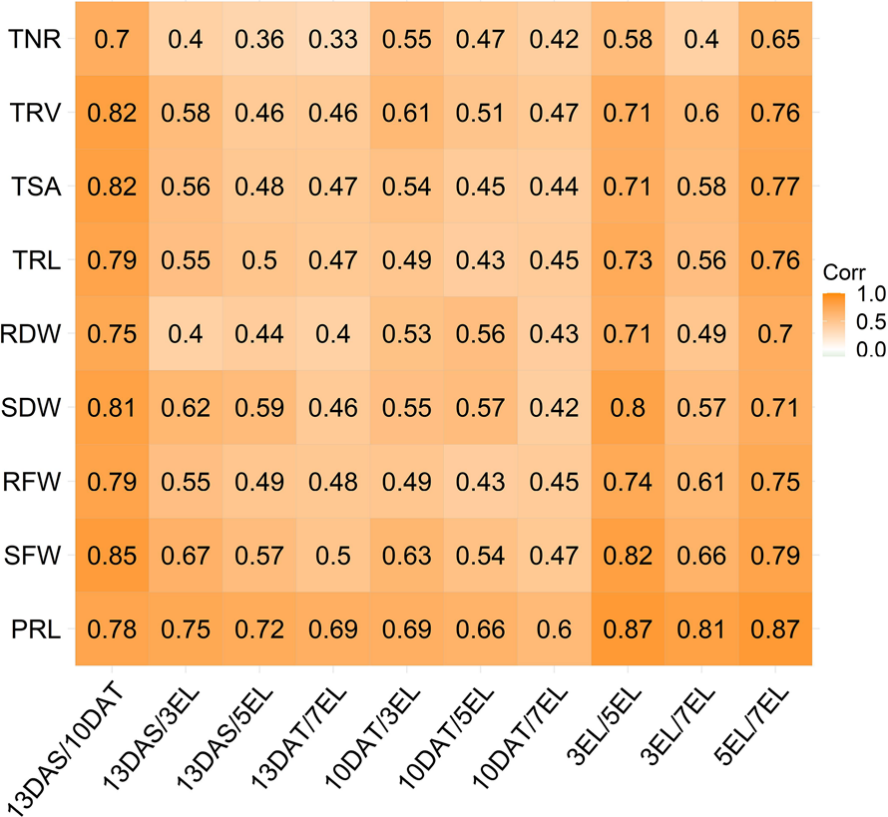
Correlations of each captured trait at five stages. Forward slash represents the correlation, for example, 13DAS/10DAT represent the correlation in the traits between 13 DAS and 10 DAT, *P* < 0.0001

**Fig. 2:**
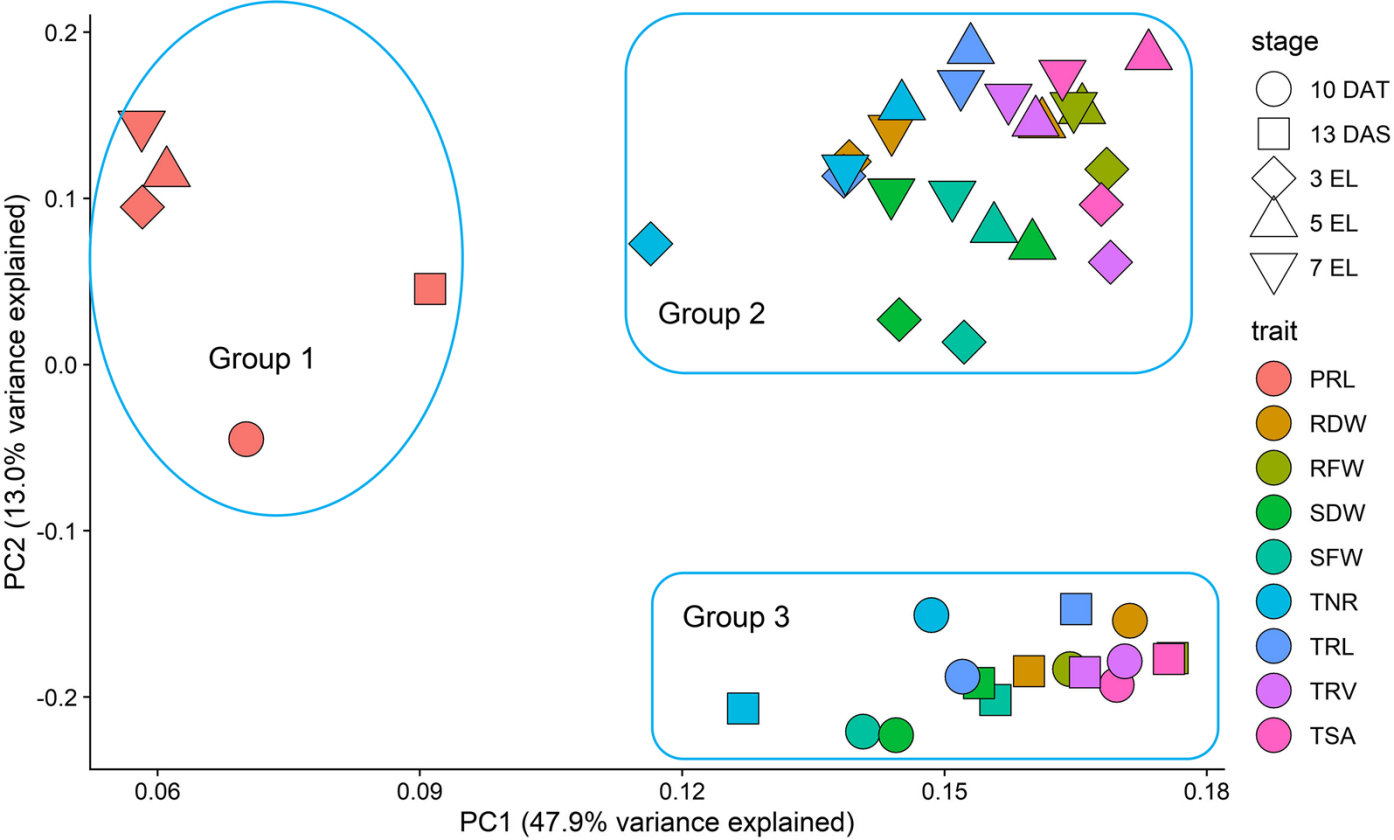
Principal component analysis (PCA) of captured traits at five stages in association population. Three obvious groups are indicated with blue circles. PRL trait was clustered into group 1. Other traits captured at 13 DAS and 10 DAT were clustered into group 3. The traits captured at 3 EL, 5 EL, and 7 EL were clustered into group 2

To examine dynamic growth patterns of roots during the vegetative stage, the hydroponic system was used for evaluating root-related traits and shoot biomass traits of 280 *B. napus* accessions which were sampled at 13 days after sowing (DAS) from the germination device and at 10 days after transplanting (10 DAT, equal to 16 DAS), three expanding leaves (3 EL), 5 EL, and 7 EL from the growth device with three biological replications for each sample, respectively (Additional file 1: Figure S1a–e). The statistics of the seven root-related traits (root fresh weight (RFW), root dry weight (RDW), primary root length (PRL), total root length (TRL), total root surface (TSA), total root volume (TRV), and total number of roots (TNR)), and two shoot biomass traits (shoot fresh weight (SFW) and shoot dry weight (SDW)) from each replication at the five sampling timepoints were listed in Additional file 2: Table S1, and the mean values of three replications are presented in Table 1. All the investigated traits showed a normal distribution or approximate normal distribution (Additional file 1: Figure S2). The coefficient of variation (CV) ranged from 15.21% to 25.66%, indicating considerable phenotypic variations for all the traits in the population (Table 1). All the traits showed a high broad-sense heritability (H^2^) at each developmental stage, ranging from 0.68 to 0.94. Furthermore, the H^2^ of all the traits was also high, ranging from 0.59 to 0.92 during the five developmental stages. For root traits, RFW, TRL, TSA, and TRV had heritability slightly higher than TNR and RDW (Table 1). We discovered significant correlations of each trait among all five stages with *r*^2^ ranging from 0.33 to 0.87 (*P* < 0.0001). In general, the highest root correlations were observed between two adjacent stages in spite of the gradually decreased correlation with the increased sampling interval, indicating that the effects of environment on these traits increased with plant development (Fig. 1). The PCA results of the traits suggested that component 1 (*X* axis, 47.9%) and component 2 (*Y* axis, 13.0%) explained the majority of genetic variation in this population (Fig. 2). With exception of PRL (Group 1), all the other traits examined at early stages (13 DAS and 10 DAT) were clustered into Group 3, whereas the traits recorded at late stages (3 EL, 5 EL, and 7 EL) were clustered into another group (Group 2). The separation of PRL and the other traits on the *X* axis indicated the substantial differences between PRL and the other traits. Traits captured at early stages (Group 3) and late stages (Group 2) were separated by the *Y* axis, but mapped to the same position on the *X* axis, suggesting the high correlations; however, a degree of specificity between the traits at early and late stages (Fig. 2). As shown in Additional file 2: Table S2, all the traits were significantly correlated with r^2^ ranging from 0.24 to 0.74 (*P* < 0.001) among the three biological replications. The results suggested that early root development traits were positively correlated with late root traits, thus suggesting that root development was a continuous process influenced by early genetic factors.

**Table 1:** Nine trait statistics of 280 accessions collected at five continuous stages

Traits	Environment	Min	Max	Mean	SD	CV (%)	*σg*^2^	*σg* × *e*^2^	*σ*^2^	H^2^	H^2^
PRL, cm	13DAS	4.85	18.48	11.22	2.14	19.06	52.74	10.95	2.45	0.93	0.93
	10DAT	8.36	27.81	17.08	3.12	18.28	74.37	19.65	6.31	0.91	
	3EL	11.23	28.16	17.87	2.96	16.58	78.47	23.98	8.87	0.90	
	5EL	11.87	27.71	18.91	2.88	15.21	74.52	19.45	8.00	0.91	
	7EL	13.03	31.30	20.09	3.10	15.44	85.20	20.93	7.79	0.92	
SFW, g	13DAS	0.21	0.94	0.52	0.11	20.95	0.129	0.020	0.009	0.94	0.65
	10DAT	0.42	2.06	1.24	0.27	21.53	0.450	0.112	0.020	0.92	
	3EL	1.22	4.58	2.95	0.56	18.97	2.794	0.816	0.207	0.90	
	5EL	3.87	17.21	10.92	1.91	17.51	32.64	8.86	2.53	0.85	
	7EL	10.20	39.97	25.12	4.40	17.53	171.56	71.56	12.56	0.87	
RFW, g	13DAS	0.031	0.152	0.084	0.020	23.23	0.003	0.001	0.000	0.93	0.67
	10DAT	0.046	0.270	0.175	0.039	22.03	0.010	0.003	0.001	0.90	
	3EL	0.218	0.839	0.487	0.097	19.84	0.083	0.026	0.008	0.90	
	5EL	0.629	2.754	1.621	0.335	20.64	1.004	0.371	0.087	0.81	
	7EL	1.24	5.42	3.07	0.63	20.45	3.52	1.45	0.286	0.87	
SDW, mg	13DAS	9.89	46.42	27.03	5.88	21.75	291.57	73.0	40.6	0.74	0.64
	10DAT	23.89	128.9	74.57	15.79	21.18	2488.4	752.8	390.3	0.84	
	3EL	61.78	250.3	160.9	32.63	20.28	9387.8	3590.5	1069.1	0.88	
	5EL	231.1	1067	659.7	117.4	17.79	123,001	53,857	12,285	0.80	
	7EL	647.8	2482.2	1632.9	306.4	18.77	825,608	434,176	77,621	0.84	
RDW, mg	13DAS	1.58	6.11	3.69	0.83	22.43	2.03	–	0.50	–	0.63
	10DAT	2.44	13.09	8.45	1.68	19.92	71.87	55.53	84.98	0.68	
	3EL	10.00	30.78	19.71	3.83	19.44	115.5	49.3	19.2	0.81	
	5EL	29.11	98.78	61.26	11.42	18.65	1169	479.3	127.7	0.76	
	7EL	59.00	294.3	143.4	33.06	23.06	8580	4926	804.3	0.82	
TRL, cm	13DAS	50.03	294.0	160.5	36.86	22.96	11,290	2049	934.16	0.93	0.71
	10DAT	142.2	772.2	457.3	104.5	22.86	31,861	11,146	2112	0.89	
	3EL	495.7	1337	778.1	139.6	17.94	172,904	43,177	15,901	0.91	
	5EL	980.3	2679	1671	297	17.77	784,719	260,737	68,229	0.89	
	7EL	1262.9	5134	2967	601	20.26	3,192,567	1,348,006	280,825	0.87	
TSA, cm^2^	13DAS	4.68	19.65	11.23	2.40	21.38	45.61	8.39	4.36	0.93	0.68
	10DAT	8.11	43.33	27.91	5.69	20.39	116.64	41.60	9.13	0.89	
	3EL	34.81	93.31	59.95	9.56	15.94	812.0	221.1	89.99	0.91	
	5EL	85.83	224.0	146.2	24.0	16.40	5090	1603	485.5	0.90	
	7EL	116.2	483.7	268.1	51.9	19.35	23,836	10,067	2025	0.87	
TRV, cm^3^	13DAS	0.025	0.119	0.063	0.014	22.20	0.002	0.000	0.000	0.93	0.64
	10DAT	0.037	0.211	0.137	0.028	20.13	0.003	0.001	0.000	0.89	
	3EL	0.185	0.570	0.374	0.063	16.98	812.0	221.1	89.99	0.91	
	5EL	0.482	1.774	1.030	0.189	18.33	0.315	0.100	0.029	0.90	
	7EL	0.859	3.639	1.947	0.402	20.63	1.427	0.584	0.112	0.87	
TNR	13DAS	60.52	233.3	121.3	26.3	21.71	11,974	4531	972	0.88	0.59
	10DAT	139.2	673.5	382.8	82.3	21.49	26,044	10,602	1977	0.87	
	3EL	284.7	924.8	521.5	93.7	17.96	77,915	22,475	8438	0.90	
	5EL	603.3	2169	1273	265.2	20.84	623,914	319,736	61,184	0.85	
	7EL	1175	5318	2759	708.0	25.66	4,394,488	2,543,179	405,276	0.83	

*Min* minimum of values in the population, *Max* maximum of values, *Mean* mean trait value, *SD* standard deviation of trait values, *CV* coefficient of variation. *σg*^2^, *σg* × *e*^2^ and *σ*^2^ estimated variance associated with the effect of genotype, genotype × environment and the residual error, respectively (*P* < 0.0001). *H*^2^ broad-sense heritability

### Persistent and stage-specific QTL clusters related to the root system are identified by GWAS

After filtering, a total of 23,542 SNPs with known physical position in the *B. napus* Darmor-bzh reference genome were selected for GWAS [34]. The distribution of the 23,542 SNP markers and LD decay on each chromosome were presented in Additional file 2: Table S3. Approximately 58.1% of the kinship coefficients between individual accessions were equal to zero, and 97.6% were less than 0.2, suggesting a weak kinship for most accessions in the natural population (Additional file 1: Figure S3).

**Fig. 3:**
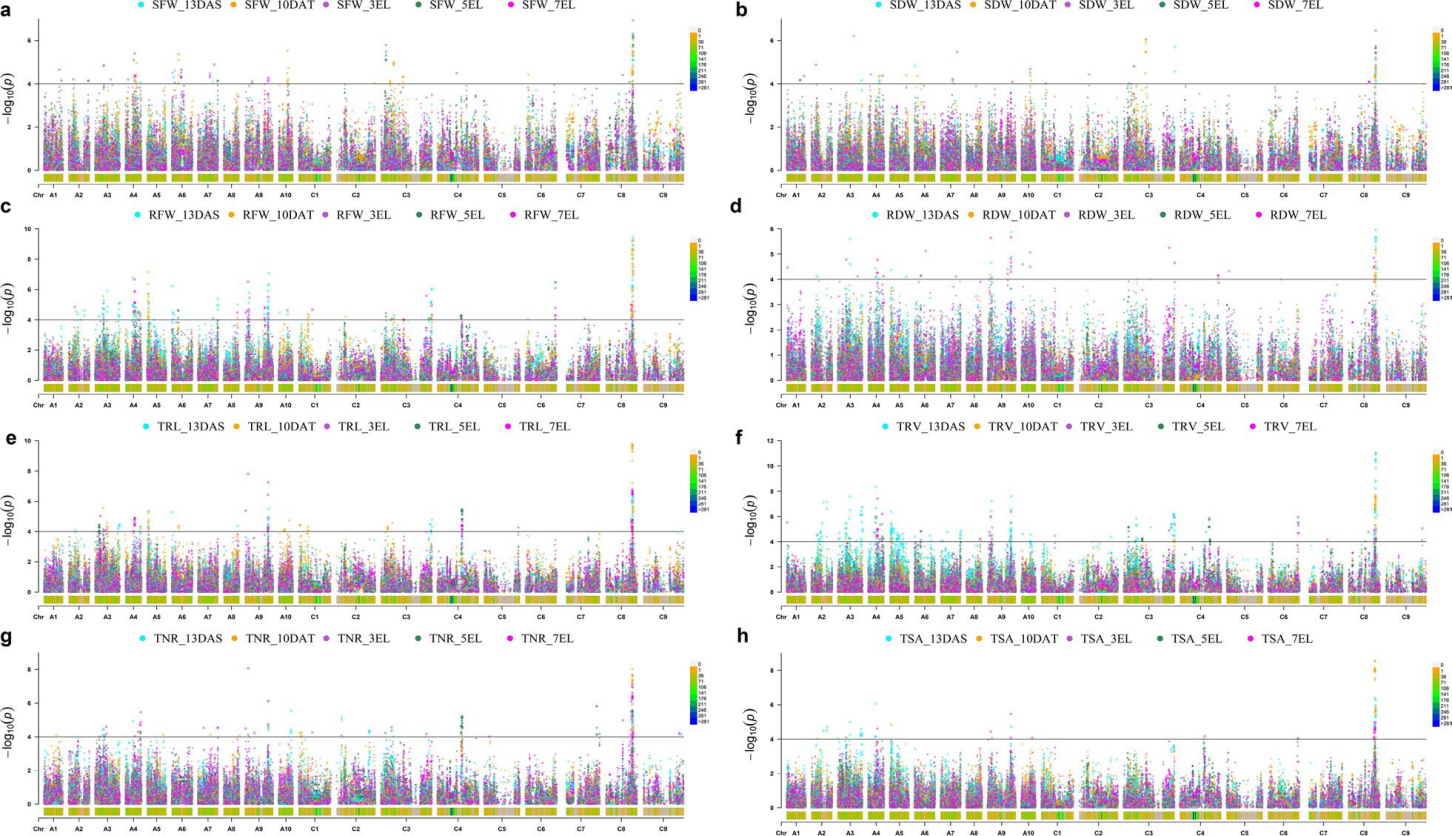
Manhattan plot of genome-wide association analysis for root and shoot-related traits at five stages. **a**–**h** Manhattan plot of genome-wide association analysis for SFW, SDW, RFW, RDW, TRL, TRV, TNR, and TSA, respectively. The different colours represent the trait-related SNPs at 13 DAS, 10 DAT, 3 EL, 5 EL, and 7 EL, respectively. The horizontal black lines indicate the significance threshold of GWAS (− log_10_^1/23,542^ = 4.37). The *x*-axis shows the 19 chromosomes (A1–A10 and C1–C9) in *B. napus*. Each chromosome is scaled by the physical chromosome length

**Fig. 4:**
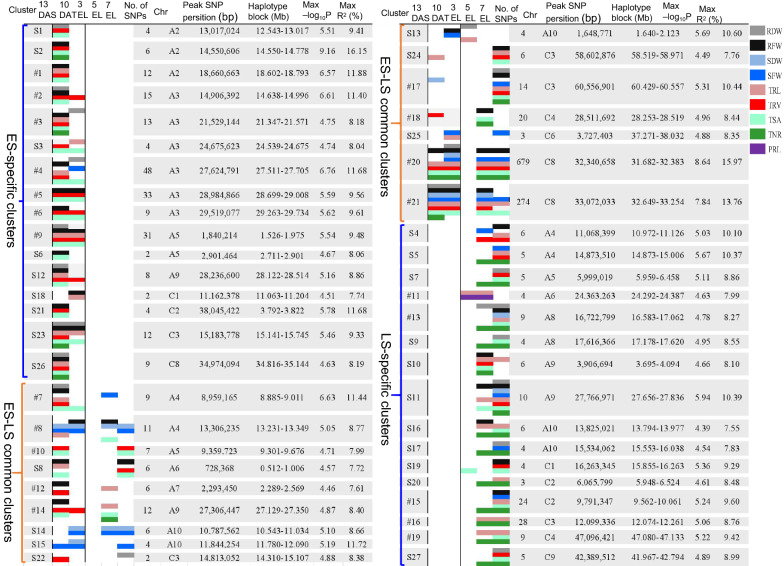
Genetic landscapes of QTL clusters obtained in this study. QTL clusters refer to significant SNPs with close proximity (within 1 Mb) and an LD of *r*^2^ > 0.2. Position, physical range, number, largest genetic variation explanation of significant SNPs of each cluster. # represents clusters with multiple SNPs. S indicates the clusters harbouring single SNPs. ES-specific clusters: early stage specific clusters, including 13 DAS and 10 DAT; LS-specific clusters: late stage specific clusters, including 3 EL, 5 EL, and 7 EL; ES–LS common clusters: early and late stage clusters, including 13 DAS, 10 DAT, 3 EL, 5 EL, and 7 EL

The 1,107 significant trait–SNP associations were detected (− log_10_^P^ > 4.37, − log_10_^1/23,542^) using the mixed linear model (MLM) for three repetitions (Additional file 2: Table S4). The manhattan plots were drawn using the best linear unbiased prediction (BLUP) values of three repetitions for all the traits to give visual GWAS results at various stages (Fig. 3). We termed the SNPs with close proximity (within 1 Mb) and an LD *r*^2^ > 0.2 as one cluster, since these SNPs were identified as the same QTL [24]. As a result, a total of 683 identified significant trait–SNP associations with 134 significant SNPs markers, and 747 suggestive trait–SNP associations (3.5 <  − log_10_^P^ ≤ 4.37) were integrated into 48 valid QTL clusters (Fig. 4, Additional file 2: Table S5). Of these 48 clusters, 21 QTL clusters contained multiple SNPs and 27 QTL clusters harboured single SNPs. The maximum genetic variation explained by these clusters ranged from 7.55% to 16.15%. We detected 28, 19, 4, 23, and 26 QTL clusters at the 13 DAS, 10 DAT, 3 EL, 5 EL, and 7 EL stages, respectively. Except 8 QTL clusters S1, S2, #1, S6, S8, S21, S26, and S18, all other clusters (40 out of 48) were detected at two or more stages. Noteworthy, two significant SNPs displaying the vast majority of trait–SNP associations for all the investigated traits except PRL on chromosome C8 were detected at multiple stages (Fig. 3; Additional file 2: Table S4). This suggested the existence of genetic factors controlling multiple root-related traits at various stages.

To reveal the genetic basis of root traits at the multiple vegetative stages, these QTL clusters were divided into three categories based on their identification stages: ES-specific clusters (early stage, 13 DAS and 10 DAT), LS-specific clusters (later stage, 3 EL, 5 EL, and 7 EL), and ES–LS common clusters. Sixteen out of the 48 QTL clusters constituted ES–LS common clusters, indicating the existence of the persistent QTL controlling root development. In addition, 16 ES-specific clusters and 16 LS-specific clusters revealed genetic mechanism in the root system at specific stages (Fig. 2; Additional file 2: Table S5). The major QTL identified in this study could be applied for improving root system architecture in rapeseed.

### Transcriptome analysis reveals dynamic root development

**Fig. 5:**
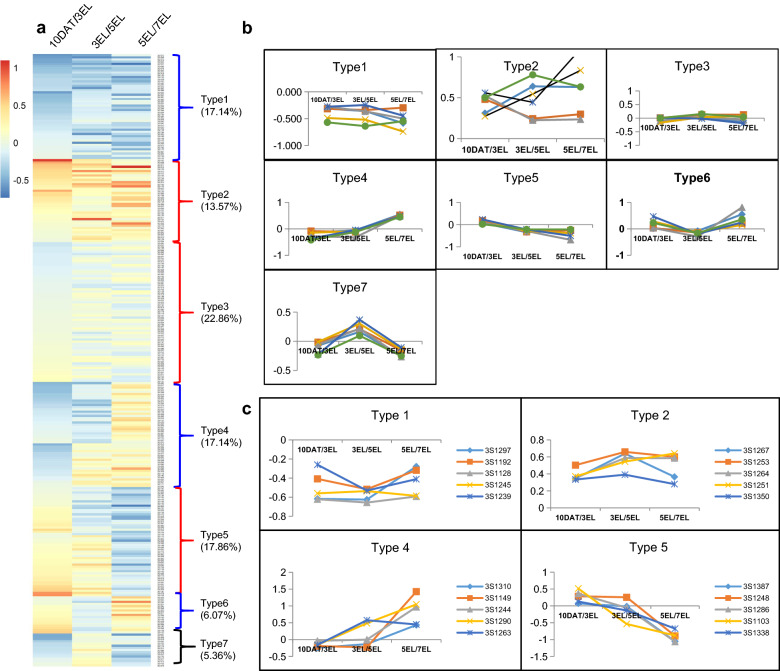
Clustering analysis of 280 accessions. **a** Hierarchical heatmap of 7 dynamic growth types of 280 accessions based on normalized GR values calculated by RFW. Red represents the GRs above the average, and green indicates the GRs below the average. **b** Diagram of 7 growth types with six accessions in each growth type. **c** Diagram of 4 transcriptome-sequenced growth types (type 1, 2, 4, and 5) with 5 accessions in each type

Clustering analysis of the 280 accessions was performed to examine the similarity and diversity of their root growth patterns. At the same the growth stage, the traits (except PRL) exhibited significant correlations (*P* < 0.0001) with each other (from 0.44 to 0.97, *P* < 0.0001; Additional file 2: Table S6), suggesting developmental relevance among these root-related traits. SFW was considered as the trait reflecting the plant growth status. RFW showed higher correlations with SFW (0.70–0.79) than with other root traits (Additional file 2: Table S6). The traits investigated at 13 DAS and 10 DAT were from different growth devices (germination device and growth device), so growth rate (GR) from 13 DAS to 10 DAT was not shown in this study. The normalized GRs were calculated by RFW to present the root dynamic growth patterns, the heatmap showed that the 280 accessions fell into seven growth types (Types 1–7) (Fig. 5a). The 38 accessions (accounting for 13.57%) belonged to growth type 1 with their GRs below the average GR from 10 DAT to 7 EL, and at least one GR less than 80% of the average. The 48 accessions (17.14%) belonged to growth type 2 with their GRs greater than the average GR from 10 DAT to 7 EL, and at least one GR greater than 120% of the average. The majority of the accessions (64, 22.86%) belonged to type 3 with a relatively stable GR ranging from 80 to 120% of the average GR from 10 DAT to 7 EL. Type 4 possessed 48 accessions (17.14%) whose GRs were below average GR from 10 DAT to 3 EL or from 10 DAT to 5 EL, but were above average from 3 to 7 EL or from 5 to 7 EL. Contrastive, type 5 consisted of 50 accessions (17.86%) whose GRs were above average GR from 10 DAT to 3 EL or from 10 DAT to 5 EL, then below average from 3 to 7 EL or from 5 to 7 EL (Fig. 5a, b). Type 6 contained 17 accessions (6.07%) with its GR changing from fast to slow, and then to fast again during the investigated stages. On the contrary, the GRs of type 7 consisting of 15 accessions (5.36%) were subjected to the change pattern of first slow, and then fast, followed by slow. Obviously, the majority of accessions fell into type 1 (with consistent slow GR) and type 2 (with consistent fast GR), and type 3 (with stable GR), indicating that genes expressed at an early stage might control root growth at the late stage with prolonged effects. In addition, type 4 and type 5 displayed obvious stage-specific changes in GRs, suggesting the existence of genes functioning at a specific stage.

The GRs of type 3 were close to average GR. The GRs of type 6 and type 7 exhibit two reverse changes during root development. Considering this, we excluded type 3, 6, and 7 in subsequent transcriptome analysis. We selected the four growth types (type 1, type 2, type 4, and type 5) for subsequent transcriptome analysis, because type 1 and type 2 had contrasting (slow and fast) GRs throughout root development stage, type 4 and type 5 exhibited opposite changing GRs at the specific stage. Five accessions from each growth type were sampled at the 10 DAT, 3 EL, 5 EL, and 7 EL stages, respectively (Fig. 5c), and were subjected to transcriptome analysis to reveal the temporal molecular mechanisms of root development. A total of more than 41 million clean reads were obtained from each library after adaptor trimming, of which, 73.47–91.59% clean reads were uniquely matched to *B. napus* reference genome (Additional file 2: Table S7). The qRT-PCR of 20 genes was performed in all the samples (Additional file 2: Table S14). The results of qRT-PCR were highly consistent with those of RNA-Seq data, suggesting the reliability of the RNA-Seq data (Additional file 1: Figure S4). The PCA of the RNA-Seq data indicated that all the four root growth types displayed an obvious separation between group 1 (10 DAT and 3 EL stage) and group 2 (5 EL and 7 EL stage) on component 1 (Additional file 1: Figure S5), indicating a change in gene expression from 3 to 5 EL during root development.

### Persistent and stage-specific mechanisms underlying root development are revealed by transcriptome analysis

**Fig. 6:**
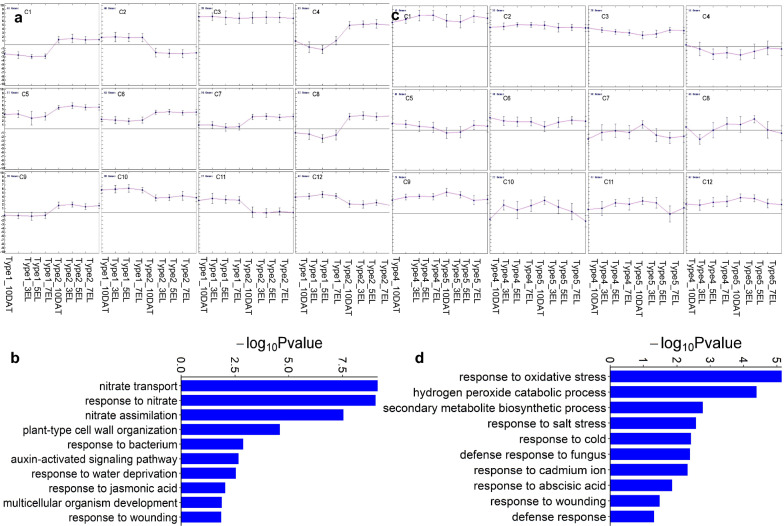
Information of persistent and stage-specific DEGs during root development in rapeseed. **a** *K*-mean clustering analysis of persistent DEGs in type 1 and type 2. The *x* axis indicates the samples at the four stages of type 1 and type 2. The *y* axis denotes the log_2_^(FPKM)^. error bars represent the min and max data. **b** GO terms enriched with the persistent DEGs in type 1 and type 2. **c** *K*-mean clustering analysis of the stage-specific expression genes in type 4 and type 5. The x axis indicates the samples at the four stages of type 4 and type 5, and the y axis denotes the log_2_^(FPKM)^. Error bars represent the min and max data. **d** GO terms enriched with the stage-specific expression genes in type 4 and type 5

To explore the persistent genetic factors during root development, the VENN analysis of the DEGs from growth type 1 vs type 2 at the four root development stages was performed. A total of 367 DEGs were found to be overlapped within the four stages (Additional file 2: Table S8). A K-means clustering analysis of these persistent DEGs showed that the expressions of these genes were stabilized among the four stages, but they exhibited significant difference between type 1 and type 2 (Fig. 6a). In addition, 35 persistent DEGs encoded transcription factors, belonging to the families of bHLH NAC, MYB, MYB_related, MADS-box, and E2F/DP. The genes *BnaAnng09810D*, *BnaA01g20660D*, *BnaC03g61210D* homologous to the MADS-box family member *ARABIDOPSIS NITRATE REGULATED 1, ANR1*, the NAC transcription factor family *PEROXIDASE 34*, *PER34*, and *bHLH25,* respectively, which have been reported to participate in root development [35–37], were expressed higher at all the stages of type 1 than type 2. The GO enrichment analysis showed that the 367 DEGs were enriched in GO terms related to energy metabolism (including acetyl-CoA biosynthetic process from pyruvate and glycolytic process) and biotic or abiotic stress (such as response to oxidative stress, hydrogen peroxide catabolic process and cold acclimation) (Fig. 6b; Additional file 2: Table S9).

Meanwhile, 485 stage-specific DEGs were identified, which exhibited lower or higher expressions at type 4 early stages than at late stages, and displayed opposite expression patterns at type 5 corresponding stages (Fig. 6c; Additional file 2: Table S10). The GO enrichment analysis revealed that these genes were significantly enriched in GO terms, such as nitrate metabolism (including nitrate transport, response to nitrate, nitrate assimilation, and cellular response to nitrogen starvation), plant-type cell wall organization, and glucosinolate catabolic process. (Fig. 6d; Additional file 2: Table S11). In this study, a total of 16 stage-specific DEGs were highly expressed at 10 DAT of type 5, and they encoded multiple transcription factors, including trihelix family genes (*PETAL LOSS* and *PTL*) and MYB_related genes (*CAPRICE* and *CPC*)*.* These transcription factors have been reported to affect root development in Arabidopsis [38, 39]. These results suggested that the biological processes, energy metabolism, and response to biotic or abiotic stress might influence the persistent root development, whereas the nitrate metabolite process might function at a specific stage during root growth. The root development was also regulated by several important transcription factors.

### Crucial candidate genes are identified by integrating GWAS, WGCNA, and differential expression analysis

**Fig. 7:**
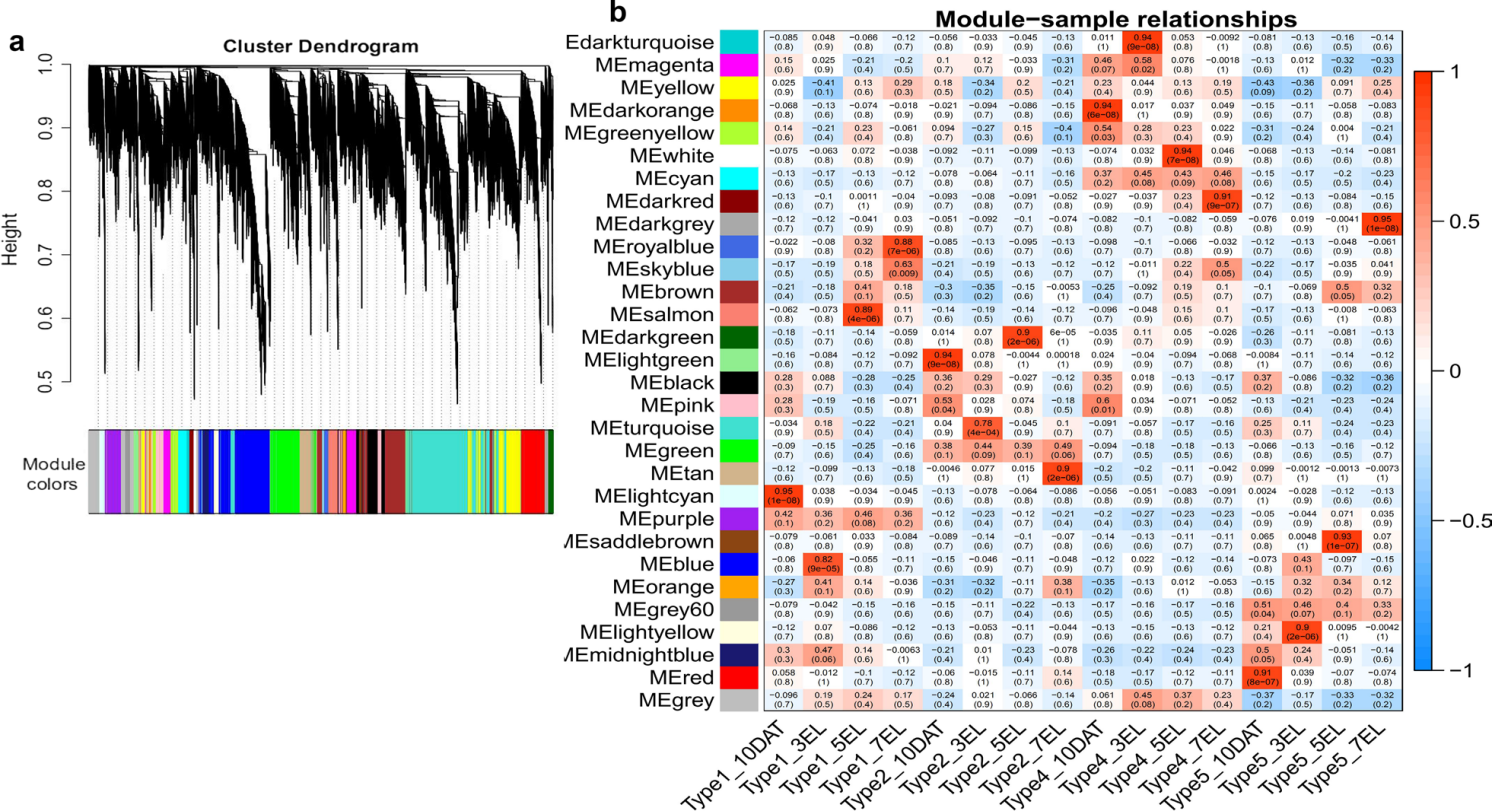
WGCNA of gene expression matrix in rapeseed. **a** Hierarchical clustering tree (dendrogram) of genes based on co-expression network analysis. **b** Module–sample association. Each row corresponds to a module labeled with a color identical to that in **a**, and each column corresponds to a sample

To investigate the gene regulatory network during root development, 26,039 DEGs from the four root growth types were used to identify co-expression gene modules by WGCNA. A total of 30 modules were identified in the dendrogram according to the correlations of genes (Fig. 7a), and the relationships between modules and samples were presented in Fig. 7b. The purple module was associated with all the stages of type 1, whereas the green module was associated with all the stages of type 2. The darkorange, darkturquoise, white, and darkred modules were significantly associated with 10 DAT, 3 EL, 5 EL and 7 EL of growth type 4, respectively, and the red, lightyellow, saddlebrown and darkgrey modules exhibited high correlations with 10 DAT, 3 EL, 5 EL and 7 EL of type 5, respectively. The heatmaps showed that the genes within one module were highly expressed in the samples highly correlated with the module (Additional file 2: Figure S6). GWAS results indicated that 2,461 genes were located in the haplotype blocks of the 48 QTL clusters (Additional file 2: Table S12). Considering a high correlation of WGCNA genes with each module (*r*^2^ > 0.85), 9 persistent and 13 stage-specific candidate genes each including 3 DEGs were screened from GWAS and WGCNA overlapped genes (Tables 2, 3). In addition, 6 GWAS and DEGs overlapped genes with correlations to the modules < 0.85 were also screened as stage-specific candidate genes (Table 3).

**Table 2:** Key persistent candidate genes identified by GWAS, WGCNA, and differential expression analysis

Gene ID	Cluster	Distance to peak SNP (Kb)	Type1_10DAT	Type1_3EL	Type1_5EL	Type1_7EL	Type2_10DAT	Type2_3EL	Type2_5EL	Type2_7EL	Gene_symbol	Description
Purple												
BnaA05g11320D^b^	S7	342.28	4.43	1.89	3.13	3.72	1.89	1.64	1.64	1.48	PCMP-E75	Pentatricopeptide repeat-containing protein
BnaA10g24040D^b^	S17	236.56	4.53	5.54	5.48	4.13	3.50	3.14	3.54	1.15	–	–
Green												
BnaA05g03210D^a^	#9	35.9	9.80	4.22	6.64	5.89	26.85	52.49	36.34	36.96	EPSIN2	Clathrin interactor EPSIN 2
BnaC02g10480D^a^	S20	50.6	1.80	0.78	0.15	0.56	16.07	42.07	16.13	31.31	SBT4.8	Subtilisin-like serine endopeptidase family protein
BnaC02g10710D^a^	S20	157.3	0.07	0.23	0.27	0.27	4.07	5.84	3.18	3.94	RID1	ROOT INITIATION DEFECTIVE 1
BnaA02g20510D^b^	S1	170.99	8.96	5.62	5.06	5.02	17.57	10.61	11.67	15.96	RSH1	Putative GTP diphosphokinase RSH1
BnaA03g52990D^b^	#4	69.3	3.36	7.90	0.89	2.10	11.78	14.47	9.05	12.04	GATA3	GATA transcription factor 3 (GATA3)
BnaC08g35330D^b^	#21	168.29	206.76	116.64	58.63	134.22	332.26	443.97	227.90	382.19	–	–
BnaC08g39040D^b^	S26	6.28	7.63	5.94	5.02	8.72	18.90	22.82	17.99	14.99	PSS1	CDP-diacylglycerol-serine O-phosphatidyltransferase 1

^a^Candidate genes overlapped by WGCNA, GWAS and DEGs

^b^Candidate genes overlapped by WGCNA and GWAS

**Table 3:** Crucial stage-specific candidate genes identified by GWAS, WGCNA, and differential expression analysis

Gene ID	Cluster	Distance to peak SNP (Kb)	Type4_10DAT	Type4_3EL	Type4_5EL	Type4_7EL	Type5_10DAT	Type5_3EL	Type5_5EL	Type5_7EL	Gene_symbol	Description
Darkorange												
BnaA05g22690D^c^	S9	409.40	12.06	2.92	3.27	1.33	1.61	0.88	7.62	0.16	–	–
BnaA08g24120D^c^	#13	238.10	9.35	4.23	2.85	4.51	1.44	2.90	5.88	6.59	FUT6	Fucosyltransferase 6
White												
BnaA03g43140D^b^	#3	106.11	1.28	1.19	3.24	1.16	0.37	0.03	0.07	0.00	FCAALL.41	Putative glycerol-3-phosphate transporter 4
BnaA03g47900D^b^	S3	34.26	0.00	0.35	2.79	0.85	0.66	0.41	0.00	0.27	–	–
BnaA09g07790D^b^	S10	107.12	0.66	0.70	2.66	0.99	0.65	0.39	0.27	0.18	DAR3	Protein DA1-related 3
BnaC08g33940D^b^	#20	118.76	1.64	3.88	12.71	4.35	2.83	0.56	1.69	0.27	REIL2	Cytoplasmic 60S subunit biogenesis factor REI1 homolog 2
Red												
BnaA03g42930D^a^	#3	32.11	2.07	4.87	1.50	2.05	13.84	1.41	0.31	1.28	–	–
BnaA09g07840D^a^	S10	89.43	2.24	3.90	1.15	3.02	12.80	5.08	1.66	1.95	PSBO1	Oxygen-evolving enhancer protein 1–1
BnaC01g22700D^a^	S19	0.06	3.35	4.12	4.70	5.38	22.74	7.92	4.62	4.68	FD3	Ferredoxin-3
BnaA03g54270D^b^	#5	252.24	0.39	0.44	0.46	0.41	4.20	1.38	0.63	0.75	–	–
BnaA05g05400D^b^	S6	114.77	22.97	21.13	26.99	30.04	58.49	33.78	26.45	37.25	PRA1B2	PRA1 family protein B2
BnaA08g24190D^b^	#13	274.89	1.63	2.80	1.19	1.59	10.52	4.34	1.44	1.20	–	–
BnaC02g14450D^b^	#15	92.11	2.23	0.00	0.86	1.19	85.14	0.04	0.55	1.62	AGP22	Arabinogalactan peptide 22
BnaC03g26110D^b^	S22	59.91	20.84	8.96	13.42	18.16	44.32	19.22	17.27	12.82	F4P13.7	Universal stress protein A-like protein
BnaA06g37280D^c^	#11	28.60	5.36	5.99	5.64	4.03	16.82	5.83	4.99	4.92	BPC5	Protein basic pentacysteine5
BnaA07g02950D^c^	#12	253.94	1.35	0.72	2.20	4.45	6.65	1.41	2.66	4.18	TIM14-3	Mitochondrial import inner membrane translocase subunit
BnaA09g07580D^c^	S10	183.41	2.15	5.44	3.24	3.15	18.16	11.78	2.75	2.20	RALFL34	Protein RALF-like 34
BnaC02g14330D^c^	#15	26.80	2.86	31.35	35.15	7.11	102.64	35.21	21.90	7.83	–	–
Lightyellow												
BnaA10g23820D^b^	S17	133.94	3.46	7.57	6.16	7.00	7.91	27.73	15.74	10.48	IPK2a	Inositol polyphosphate multikinase alpha

^a^Candidate gene overlapped by WGCNA, GWAS and DEGs

^b^Candidate gene overlapped by WGCNA and GWAS

^c^Candidate gene overlapped by GWAS and DEGs

**Fig. 8:**
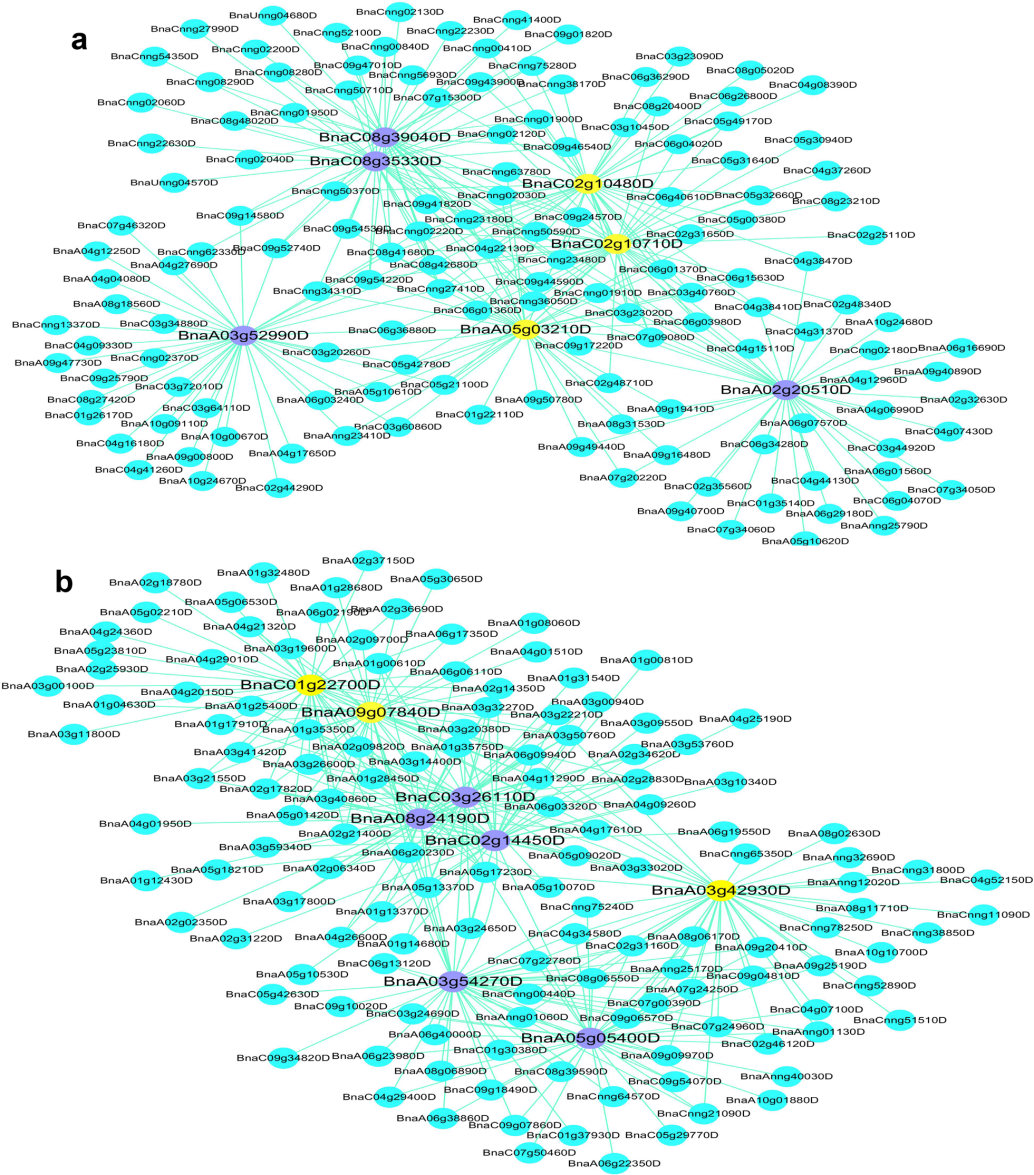
Networks of genes in green and red modules.** a**, **b** Correlation networks in green and red modules, respectively. Yellow colour in the network indicates the candidate genes overlapped by GWAS, WGCNA and differential expression analysis, and the purple colour in the network indicates the candidate genes overlapped by GWAS and WGCNA

Among the nine persistent candidate genes, two and seven genes with high correlations to the purple and green modules were highly expressed at all the stages of type 1 and type 2, respectively (Table 2). Four genes in the green module were located less than 100 kb away from the peak SNPs, including *BnaA05g03210D*, *BnaC02g10480D*, *BnaA03g52990D*, and *BnaC08g39040D* which were 35.9 Kb, 50.6 Kb, 69.3 Kb, and 6.3 Kb apart from the peak SNPs of #4, #9, S20, and S26 (Table 2). *BnaA03g52990D* encodes the GATA transcription factor, whose homolog influences root development by affecting auxin level and cell division in Arabidopsis [40]. Two genes *BnaC02g10710D* and *BnaA05g03210D* exhibited high correlation with *BnaA03g52990D* (Fig. 8a). *ROOT INITIATION DEFECTIVE 1*, *RID1* (the homolog of *BnaC02g10710D*) has been reported to function in root apical meristem and root morphogenesis in Arabidopsis [41]. Furthermore, three persistent DEGs, *BnaC02g10480D*, *BnaC02g10710D*, and *BnaA05g03210D*, displayed high correlations to each other in the green module (Fig. 8a).

Of the 19 stage-specific candidate genes, *BnaA03g47900D* in the white module and seven genes *BnaA03g42930D*, *BnaA09g07840D*, *BnaC01g22700D*, *BnaC02g14450D*, *BnaC03g26110D*, *BnaA06g37280D,* and *BnaC02g14330D* in the red module were located less than 100 kb away from the peak SNPs (Table 3). Especially, *BnaC01g22700D* encoding ferredoxin-3 protein was 0.06 kb apart from the peak SNP of S19 QTL cluster. Our data indicated that *PROTEIN BASIC PENTACYSTEINE5, BPC5* (the homologs of *BnaA06g37280D*) was located 28.6 kb away from the peak SNP of #11, and in previous study, *BPC5* has been found to promote lateral root growth in Arabidopsis [42]. Our WGCNA, stage-specific DEG analysis, and GWAS results indicated that *BnaA03g42930D*, *BnaA09g07840D,* and *BnaC01g22700D* were detected and were highly expressed at 10 DAT of type 5 (Table 3), and that these three genes were highly correlated with another three closely linked genes *BnaA08g24190D*, *BnaC02g14450D,* and *BnaC03g26110D* (Fig. 8b).

In the present study, four candidate genes were screened as crucial persistent genetic factors and eight as stage-specific genetic factors with less than 100 kb physical distances from the peak SNPs in *B. napus*. Furthermore, homologs of three candidate genes (*BnaA03g52990D*, *BnaA06g37280D,* and *BnaA09g07580D*) have been reported to regulate root development in previous studies. The results showed that the method of screening candidate genes by combining GWAS, WGCNA, and differential expression analysis was effective.

## Discussion

### Two types (persistent and stage-specific) of temporal genetic factors controlling root development in *B. napus*

Recent advances in high-resolution imaging of root growth have indicated that the root system was determined by continuous spatial and temporal growth [11, 43–45]. Consistent with the previous report [11], our phenotypic correlation analyses and root growth dynamics study revealed two types (persistent and stage-specific) of temporal genetic factors controlling root development in *B. napus*. Furthermore, the persistent and stage-specific genetic factors were verified by our identified QTL clusters and DEGs (Fig. 2; Additional file 2: Table S5). Our dynamic QTL analysis results were in line with the previous reports on several dynamic traits at different developmental stages in Arabidopsis, barley, wheat, upland cotton, maize, and *B. napus* [11, 14, 46–55]. For example, 35 dynamic conditional QTL which can enhance the number of roots were detected at different root development stages in upland cotton, suggesting the dynamic development of roots [46].

Furthermore, the peak SNPs of 18 QTL clusters in this study were co-localized in the identical haplotype blocks of the 27 previously reported significant SNPs related to root traits at low or sufficient phosphorus conditions (Additional file 2: Table S13) [14]. Clusters S10, S20, #15, and S25 were also co-localized with previously identified QTL (qcA09-1, qcC02-2, qcC02-2, and uqPRLC06) related to root surface area (RSA) trait, respectively [11, 14]. Our results provided useful QTL and the major QTL can be used for marker-assisted selection of root traits in rapeseed.

### Possible regulatory pathways of persistent and stage-specific genetic factors related to root development

Root growth, as a complex process, is determined by the interaction of many genes. Some genes play a persistent role during root development, whereas others function at a specific stage. In this study, we identified 367 persistent DEGs from growth type 1 vs type 2 controlling root development in rapeseed. Three persistent DEGs enriched in acetyl-CoA biosynthetic process were homologs of *PYRUVATE DEHYDROGENASE E1, ALPHA* in *A. thaliana* affecting polar auxin transport during root development [56]. Oxidative stress response is a general response of living organisms to biotic or abiotic stress [57]. Ten out of the 14 persistent DEGs (Additional file 2: Table S8) enriched in oxidative stress encoded 10 proteins PEROXIDASE 34 (PRX34), CYP709B3, CYP87A3, CYP78A6, PEROXIDASE 3 (PER3), PER34, PER44, PER71, CATALASE-2 (CAT2), and ALPHA-DIOXYGENASE 1 (DOX1), and these genes have been reported to act as regulators in root development in Arabidopsis [58–62]. For example, *PRX34* mediated H_2_O_2_ generation and increased Ca^2+^ flux from the cytosol of *Atmpk6* root cells to inhibit root elongation [63]. *CYP709B* subfamily was involved in cytokinin metabolism and signaling in roots [58]. These results suggested that biological processes, such as energy metabolism and biotic or abiotic stress response, especially oxidative stress response might act as the major molecule mechanisms influencing persistent root development.

NO^3−^ and nitrate metabolites can serve as regulatory signals to control root system architecture [64]. Three stage-specific DEGs (Additional file 2: Table S10) homologous to *NRT1/NPF6.3* not only regulated auxin biosynthesis to promote LR primordia emergence, but also repressed LR development by promoting auxin transport at low nitrate in Arabidopsis [65]. *High-affinity nitrate transporter 2.1* (*NRT2.1*) homologous to our four stage-specific DEGs has been reported to play an essential role in root nitrate uptake (Additional file 2: Table S10) [66]. The 485 stage-specific DEGs were found to be enriched in GO terms, such as nitrate transport, response to nitrate, nitrate assimilation, and glucosinolate catabolic process (Fig. 6; Additional file 2: Table S11). Furthermore, some previous studies have reported that the glucosinolate accumulation can restrain root growth and development [67–71]. Defense metabolite Allyl-glucosinolates (allyl-GSL) have been reported to affect Arabidopsis root development through three different catabolic products [72]. *AtTGG4* and *AtTGG5* homologous to two genes, *BnaA08g01990D* and *BnaC06g08840D*, enriching in the glucosinolate catabolic process have been reported to regulate root growth and play a part in flood tolerance in Arabidopsis [73]. The above results suggested that nitrate metabolism process and glucosinolate catabolic process might mainly regulate the stage-specific root development.

### Efficient discovery of candidate genes by combining GWAS, WGCNA, and differential expression analysis

Combination of GWAS, WGCNA, and differential expression analysis has been reported as an efficient way to acquire crucial genes in maize, rice, soybean, carrot, and other crops [9, 15, 32, 33]. We identified four persistent and eight stage-specific crucial candidate genes related to root development by integrating GWAS, WGCNA and differential expression analysis in rapeseed.

Four crucial persistent candidate genes *BnaA03g52990D*, *BnaA05g03210D*, *BnaC02g10480D, BnaC08g39040D* in the green module displayed high correlations to each other (Fig. 8), and two genes, *BnaA03g52990D* and *BnaC02g10710D* in green module were homologous to *ATGATA3* and *ATRID1* which have been reported to function in root development in Arabidopsis [40, 41]. Our identified *ATRID1* had similar function with *SRD2* which affected LR morphogenesis by reducing the level of auxin efflux facilitator (PIN) in *A. thaliana* [41, 74]. Furthermore, the homologs of other genes in the green module, *PROTEIN PHOSPHATASE 2C* (*AIP1*), *REPLICATION PROTEIN A SUBUNIT B* (*RPA1B*), *HISTIDINE KINASE 3* (*AHK3*), *POLYADENYLATE-BINDING PROTEIN 2* (*PAB2*), have also been reported to affect root development by regulating phytohormone or promoting cell elongation [75–78]. All these results indicated that the crucial persistent candidate genes in the green module might have similar functions during root development.

Seven out of eight stage-specific crucial candidate genes were in the red module, of which *BnaA06g37280D* and *BnaA09g07580D* were homologous to *BPC5* and *RALFL34* reported to promote LR development by inhibiting the abscisic acid insensitive 4 expression and activating *PIN1* level in Arabidopsis [42, 79]. Moreover, the red module included several function-known genes involved in root development, such as *BnaA08g06170D* and *BnaC08g06550D* which were homologous to *AtSMAP1* reported to modulate root development by interacting with 2,4-Dichlorophenoxyacetic acid [80]. The results above further demonstrated that these seven candidate genes played significant roles in root growth.

The candidate genes and dynamic QTL identified in this study can serve as exploitable resources to broaden our research on molecular mechanism of root development. More studies are needed to further analyze these candidate genes and validate their functions.

## Conclusions

Rapeseed provides not only edible vegetable oil for human consumption, but also an important source for biofuel production. To construct excellent root system by genetic improvement is conducive to improve rapeseed productivity. The seven dynamic patterns of root growth rates and 16 persistent and 32 stage-specific quantitative trait loci (QTL) clusters which were obtained by GWAS supported the existence of two types of QTL (persistent and stage-specific) controlling root growth at specific or multiple developmental stages, respectively. Total of 367 identified persistent DEGs were enriched in energy metabolism and biotic or abiotic stress. Whereas 485 stage-specific DEGs were enriched in nitrogen metabolism. By integrating GWAS, WGCNA, and differential expression analysis, we identified four candidate genes as crucial persistent genetic factors and eight as stage-specific genes. Among these, three candidate genes (*BnaA03g52990D*, *BnaA06g37280D*, and *BnaA09g07580D*) had been reported to regulate root development in previous studies, supporting the validity of this method to obtain candidate genes. Our results provide new insights and useful candidate QTLs/genes into the temporal genetic mechanisms of root growth in rapeseed.

## Methods

### Plant materials and growth conditions

The natural population used in this study consisted of 280 *B. napus* lines, including 156 semi-winter accessions, 86 spring accessions and 38 winter accessions. A total of the 280 rapeseed germplasm accessions were collected, including 222 accessions from the Yangtze River of China, 23 from northwestern China, 16 from Europe, 14 from Australia, and 5 from other places or unknown origins. All the accessions were strictly self-crossed.

The previously reported hydroponic system was used for the root-related trait evaluation of the 280 *B. napus* accessions [11]. Briefly, uniform and stout rapeseed seeds were placed on the medical gauze of the germination device for 2 days in the dark, then they grew in the light (180 μmol photons m^−2^ s^−1^) for 4 d in a greenhouse (60–80% relative humidity) under 16/8 h day/night cycles at 24 °C (Additional file 1: Figure S1f, g). A quarter of modified Hoagland’s nutrient solution was filled into germination device to retain moisture and provide nutrients for seed germination [81]. Six days after sowing, uniform seedlings were transferred to the growth device containing 1/4 Hoagland’s solution. The 1/4 solution was replaced with a 1/2 solution, and then with a 100% solution once a week until harvesting.

### Phenotypic evaluation of association panel

The accessions from the natural population were completely randomly grown and evaluated with three replications. In each replication, three uniform plants per accession were collected from the germination device at 13 days after sowing (DAS). At 6 DAS, 24 plants of an accession were transplanted to one growth device (Additional file 1: Figure S1h). Then three plants per accession were sampled from the growth device at four timepoints, namely, 10 days after transplanting (10 DAT, equal to 16 DAS), three expanding leaves (3 EL), 5 EL, and 7 EL, respectively. In total, 12,600 plants (280 accessions × 3 replicates × 3 plants × 5 timepoints) were sampled. Once the plants were sampled, shoot fresh weight (SFW), root fresh weight (RFW), and primary root length (PRL) were measured manually. The intact roots in a transparent box full of water were scanned with the root scanner (EPSON, 11000XL). The obtained high-resolution root images were analyzed using WinRHIZO-Pro software (Regent Instruments, QC, Canada) to determine total root length (TRL), total root surface (TSA), total root volume (TRV), and total number of roots (TNR). Subsequently, shoot and root samples were dehydrated at 65 °C for a week to determine shoot dry weight (SDW) and root dry weight (RDW).

### Data analysis

The variance and correlation analyses of the investigated traits were performed using the software SAS 9.2. The broad-sense heritability was calculated using the formula reported by Liu et al. [24]. The principal component analysis (PCA) of all the investigated traits were conducted by the software SAS 9.2. The first step of PCA was to obtain the correlation matrix between different traits, then the dimensionality reduction was performed to obtain eight principal components, and PC1 and PC2 were plotted by R. According to previous reported method [11], the growth rate (GR) of accession sample was calculated as the root fresh weight (RFW) value at the late stage minus that at the early stage, and then divided by the growing days. GR of an accession was normalized according to the following formula. Normalized GR = (GRg − GRp)/GRp In the formula, GRg represented the GR of a genotype, and GRp was the average GR of the population of 280 accessions which were clustered in terms of the normalized GR using MeV_4_9_0 software (http://mev.ro/en/).

### Population structure, relative kinship, and association analysis

The *Brassica* 60 K Illumina^®^ Infinium consortium SNP array [82] (http://www.illumina.com/technology/beadarray-technology/infinium-hd-assay.html) was used for accessions genotype. SNP data were analyzed using Illumina BeadStudio genotyping software (http://www.illumina.com/) with parameters set as a missing rate ≤ 0.2, heterozygous rate ≤ 0.2, and minor allele frequency (MAF) > 0.05. BLAST was performed to search the probe sequences of these SNPs against the *B.napus* Darmor-bzh reference genome [34] with an threshold of e^−10^. SNPs with merely one matched position in reference genome were used for further analysis. The population structure and relative kinship of the 280 *B. napus* accessions were analyzed using STRUCTURE v. 2.3.4 and SPAGeDi software, respectively [83]. The linkage disequilibrium (LD) decay between all SNPs was assessed by TASSEL 4.0 [84]. The trait–SNP association was analyzed using mixed linear model (MLM) for both the single repetition and the BLUP [85]. Marker haplotypes at each associated locus were identified using the four-gamete rule with Haploview software [86].

### Transcriptome sequencing and analysis

Five accessions from each of the four growth types (type 1, type 2, type 4, and type 5, detailed information presented in “Result” section) with contrasting GRs were selected and replanted for further transcriptome analysis. Total roots of three plants for each accession were sampled at four timepoints (10 DAT, 3 EL, 5 EL, and 7 EL) with two biological replications. Samples of the five accessions within one growth type at each sampling timepoint with the same weight were mixed as a single sample. A total of 32 obtained samples were fully mixed for total RNA extraction with the IRIzol reagent (Invitrogen, USA). Sequencing library construction and Illumina sequencing were performed by the Oebiotech Company in Shanghai, China using an Illumina HiSeq™ 2500 platform. The raw reads with 150 paired-end base pair (bp) were filtered and aligned as previously reported [25]. The raw data were submitted into database of the National Center for Biotechnology Information Sequence Read Archive (SRA; http://www.ncbi.nlm.nih.gov/sra) (Accession No. PRJNA714285).

The clean reads were mapped to the *B.napus* Darmor-bzh reference genome [34] (http://www.genoscope.cns.fr/brassicanapus/data/) using Hisat2. The gene expression levels were expressed as FPKM (fragments per kilobase per million reads) value. The PCA of the gene expressions was performed using the PCAtools package in R. The WGCNA was conducted using the WGCNA package in R [87]. *P* ≤ 0.05 for the false discovery rate (FDR) and |log_2_^ratio^|≥ 1 were used as criteria to identify DEGs with the DESeq package in R. The *k*-mean clustering was performed by MeV_4_9_0 software. Gene ontology (GO) enrichment analysis was performed using the ClusterProfiler package in R.

### Real-time reverse transcription PCR

Quantitative real-time PCR (qRT-PCR) of 20 genes randomly selected from the DEGs was performed to verify the accuracy of RNA-seq data. The primer sequences were presented in Additional file 2: Table S14. The SYBR qPCR Master Mix (Vazyme) was used for qRT-PCR analysis with the CFX96 (BIO-RAD). Three technical replications were performed for each sample. The *B. napus ACTIN2* was used as an internal control to compute the relative expression of target genes by the 2^−ΔΔCT^ method.

## Supplementary Information

**Additional file 1: Figure S1 **Phenotype of plants at different stages in *B. napus*. (**a**–**e**) Plants at 13 DAS, 10 DAT, 3 EL, 5 EL, and 7 EL, respectively. **(f**) Materials sowed on germination device. (**g**) Plants in germination device 6 days after sowing. (**h**) Plants in growth device. Scale bars = 3 cm (**a**–**e**), 4 cm (**f, g**), and 8 cm (**h**). **Figure S2 **Frequency distribution of root-related traits and shoot-related traits at five stages.** (a**–**i**) Frequency distribution of SFW, RFW, SDW, RDW, TNR, PRL, TRL, TSA, and TRV at the five stages (13 DAS, 10 DAT, 3 EL, 5 EL, and 7 EL), respectively. **Figure S3 **Analysis of population structure and kinships of 280 *B. napus* accessions (**a**) Log-likelihood data of possible clusters, K: from 1 to 10. (**b**) Distribution of pairwise relative kinship. (**c**) Population structure of 280 accessions. **Figure S4 **Positive correlation between RNA-seq data and qRT-PCR data.** Figure S5** Principal component analysis of the transcriptome sequencing data.** Figure S6** Heatmap of module eigengenes obtained by WGCNA**. (a-l**) Heatmaps of the expression profile of eigengenes in the purple, green, black, brown, darkorange, darkturquoise, white, darkred, red, lightyellow, saddlebrown and darkgrey modules, respectively.

**Additional file 2: Table S1** Trait statistics collected at the five stages of each repetition. **Table S2** Correlations in each captured trait among replication at five stages. **Table S3** Summary of SNPs and LD decay on 19 chromosomes of *B. napus.* **Table S4** Detailed information on trait-related significant SNPs identified by GWAS. **Table S5** Detailed information on 48 valid QTL clusters. **Table S6** Correlations among root-related traits at each examined stage. **Table S7** RNA-Seq statistics of four growth types against *B. napus* reference genome. **Table S8** FPKM of persistent DEGs. **Table S9** GO enrichment results of persistent DEGs. **Table S10** FPKM of stage-specific DEGs. **Table S11** GO enrichment results of stage-specific expressed genes. **Table S12** FPKM of genes located in haplotype blocks on 48 QTL clusters. **Table S13** Information on peak SNPs overlapped with SNPs reported by Wang et al. (2017). **Table S14** Primers used in this study.

## Data Availability

The raw sequence data have been deposited in the National Center for Biotechnology Information Sequence Read Archive (http://www.ncbi.nlm.nih.gov/sra/) under Accession number PRJNA714285. All other relevant data during this study are included in the manuscript and additional files.

## Abbreviations

13 DAS: 13 Days after sowing
10 DAT: 10 Days after transferring
3 EL: Three expending leaves
5 EL: Five expending leaves
7 EL: Seven expending leaves
PRL: Primary root length
RDW: Root dry weight
RFW: Root fresh weight
SDW: Shoot dry weight
SFW: Shoot fresh weight
TNR: Total root number
TRL: Total root length
TRV: Total root volume
TSA: Total root surface area
LR: Lateral root
GR: Growth rate
GWAS: Genome-wide association study
SNP: Single nucleotide polymorphism
MAF: Minor allele frequency
DEGs: Differentially expressed genes
QTL: Quantitative trait loci
qRT-PCR: Quantitative real-time PCR
RNA-seq: RNA sequencing
FPKM: Fragments per kilobase of exon per million reads mapped
CV: Coefficient of variation
H^2^: Broad-sense heritability
BLUP: The best linear unbiased prediction
