# Supplementary figures and images for: Genome-wide association study and transcriptome analysis reveal key genes affecting root growth dynamics in rapeseed
Source: Biotechnol Biofuels. 2021 Sep 10;14:178. doi: 10.1186/s13068-021-02032-7 (PMC8431925; doi:10.1186/s13068-021-02032-7)

**Figure S1**

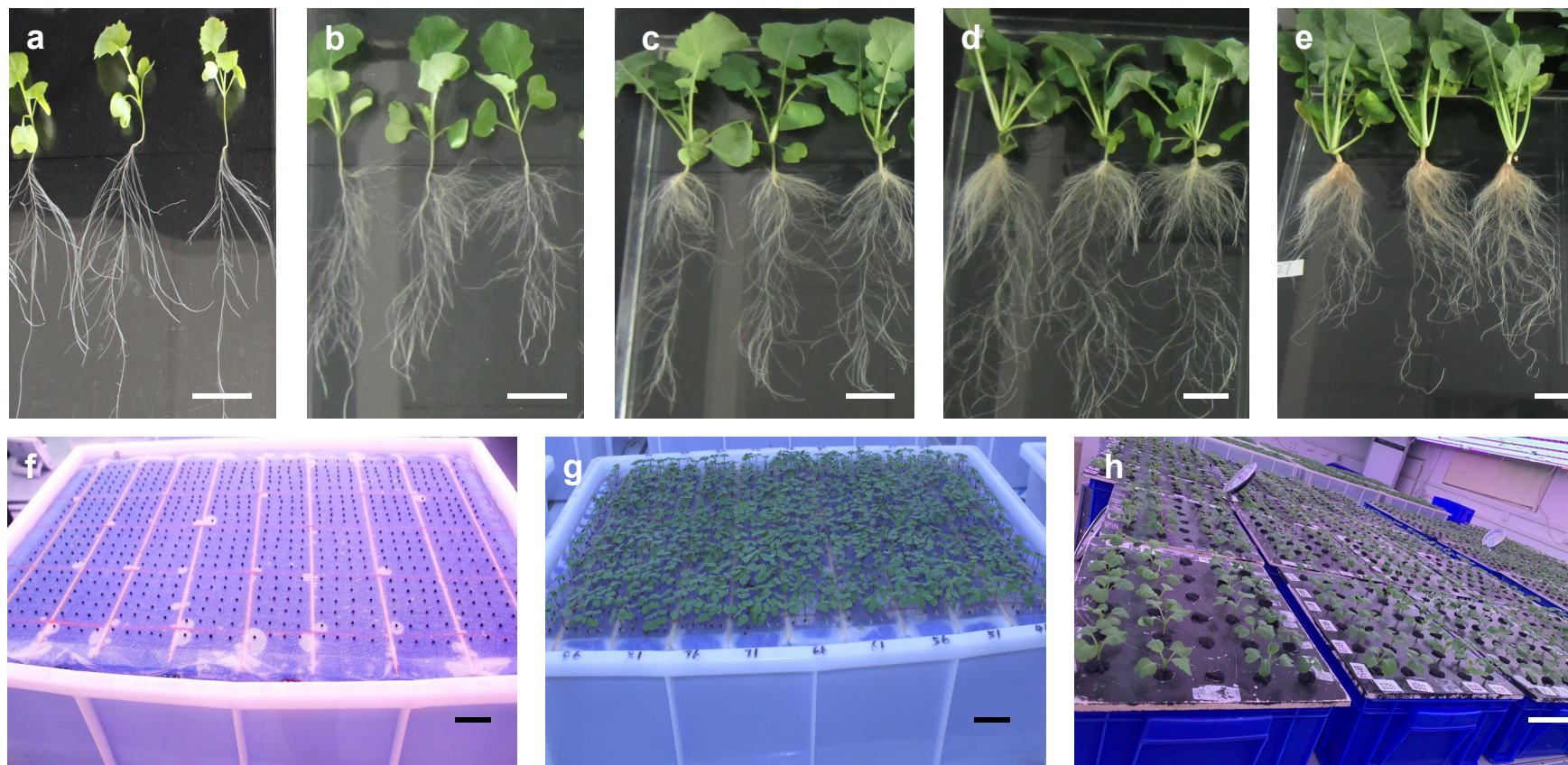

# Figure S2

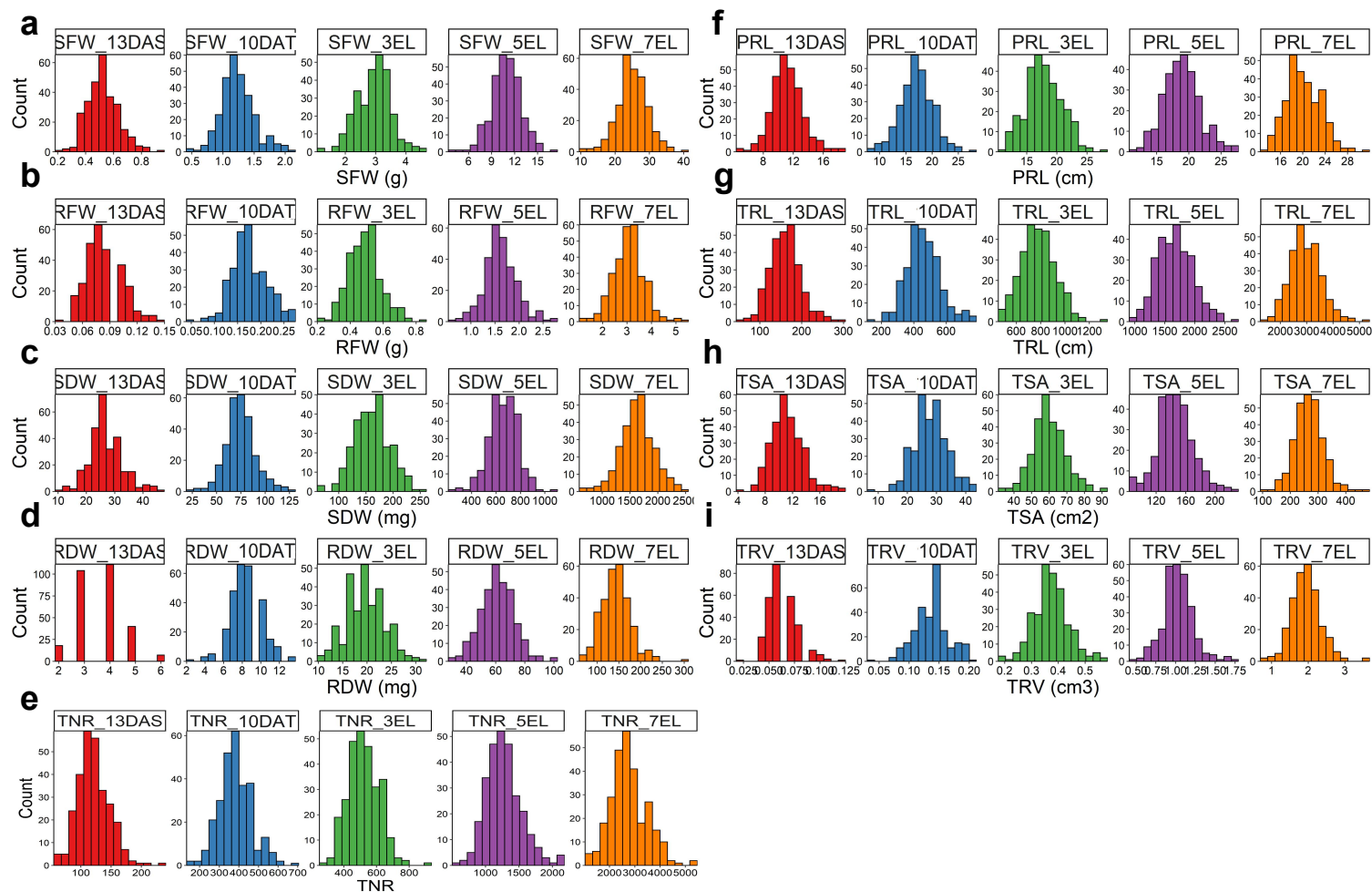

**Figure S3**

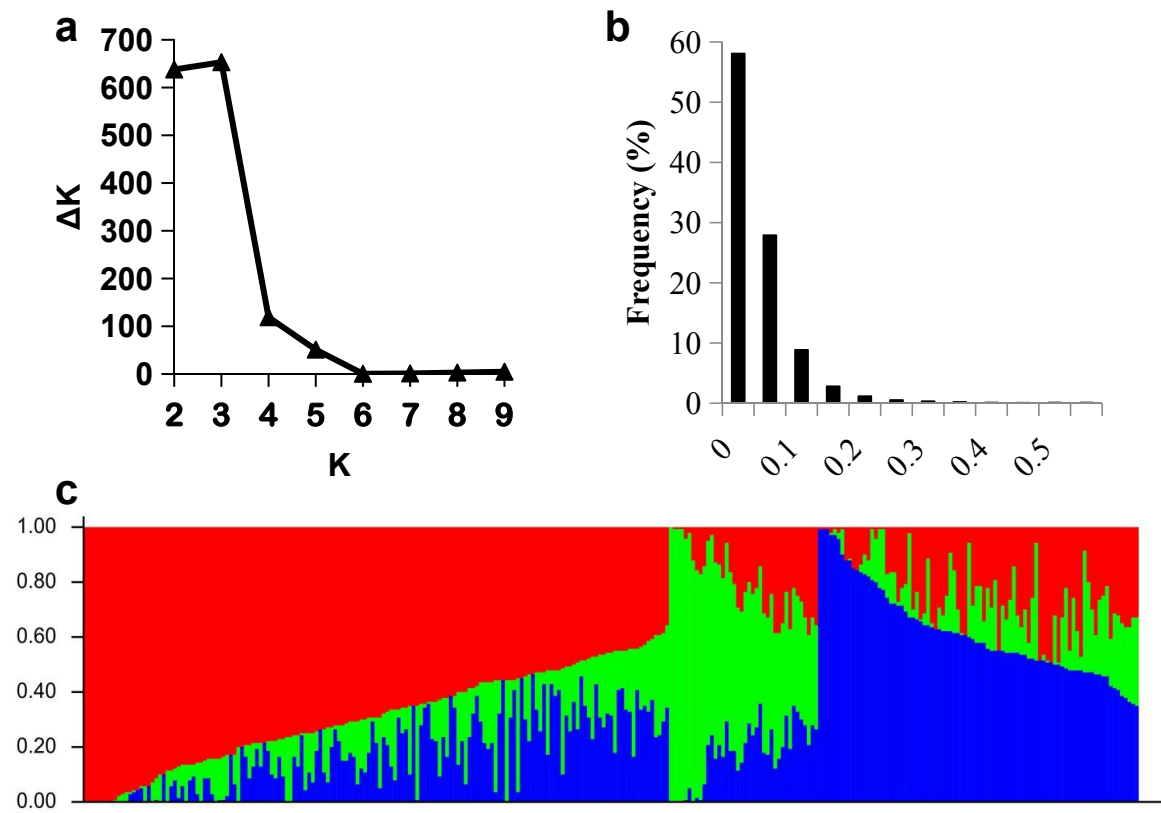

**Figure S4**

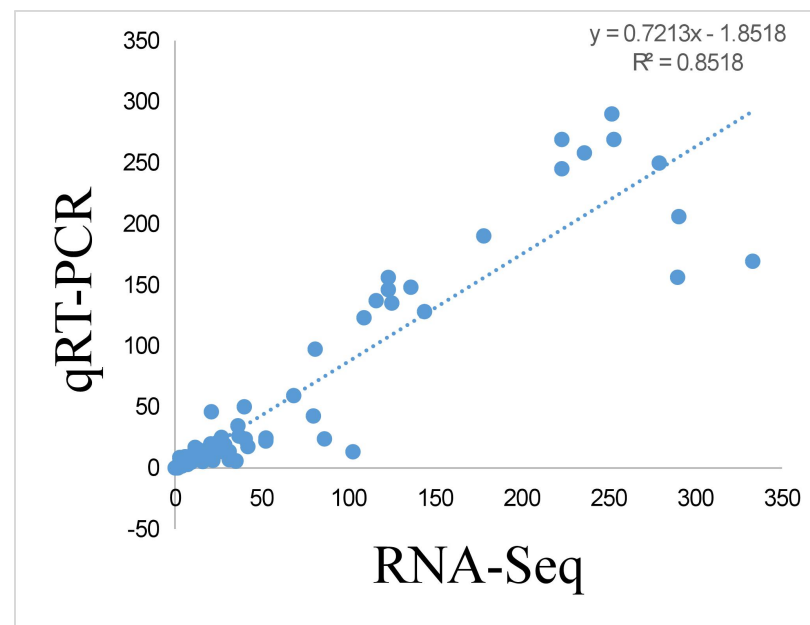

**Figure S5**

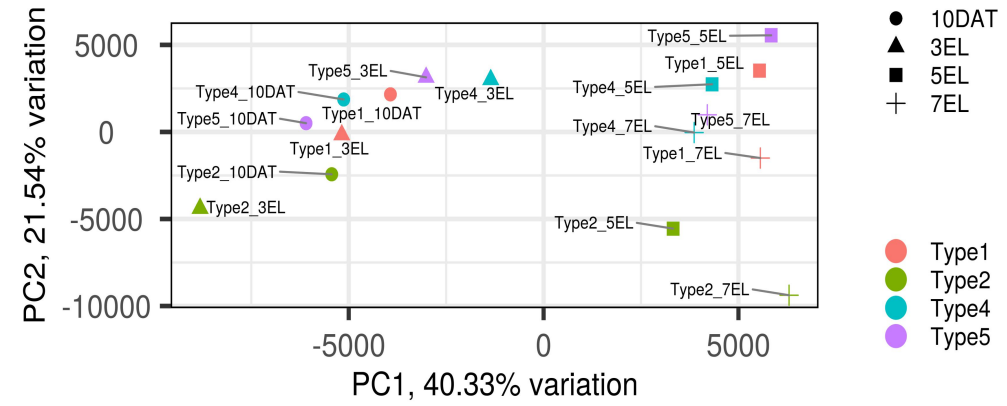

Figure S6

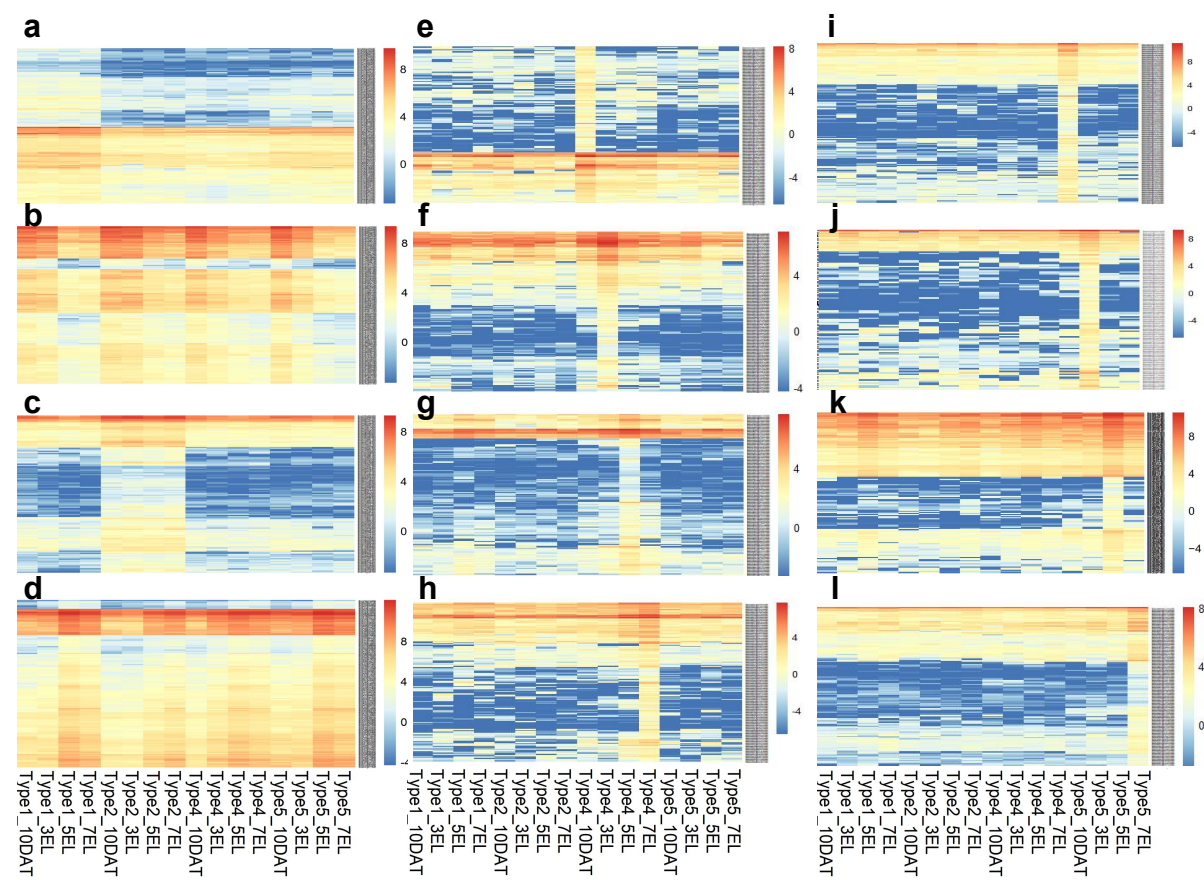

Supplement: Supplementary file 1 — Additional file 1: Figure S1 Phenotype of plants at different stages in B. napus. (a–e) Plants at 13 DAS, 10 DAT, 3 EL, 5 EL, and 7 EL, respectively. (f) Materials sowed on germination device. (g) Plants in germination device 6 days after sowing. (h) Plants in growth device. Scale bars = 3 cm (a–e), 4 cm (f, g), and 8 cm (h). Figure S2 Frequency distribution of root-related traits and shoot-related traits at five stages. (a–i) Frequency distribution of SFW, RFW, SDW, RDW, TNR, PRL, TRL, TSA, and TRV at the five stages (13 DAS, 10 DAT, 3 EL, 5 EL, and 7 EL), respectively. Figure S3 Analysis of population structure and kinships of 280 B. napus accessions (a) Log-likelihood data of possible clusters, K: from 1 to 10. (b) Distribution of pairwise relative kinship. (c) Population structure of 280 accessions. Figure S4 Positive correlation between RNA-seq data and qRT-PCR data. Figure S5 Principal component analysis of the transcriptome sequencing data. Figure S6 Heatmap of module eigengenes obtained by WGCNA. (a-l) Heatmaps of the expression profile of eigengenes in the purple, green, black, brown, darkorange, darkturquoise, white, darkred, red, lightyellow, saddlebrown and darkgrey modules, respectively. [file 13068_2021_2032_MOESM1_ESM.pdf]
